# Segmentation model of soft tissue sarcoma based on self-supervised learning

**DOI:** 10.3389/fonc.2024.1247396

**Published:** 2024-07-01

**Authors:** Minting Zheng, Chenhua Guo, Yifeng Zhu, Xiaoming Gang, Chongyang Fu, Shaowu Wang

**Affiliations:** ^1^ Department of Radiology, The Second Affiliated Hospital of Dalian Medical University, Dalian, China; ^2^ School of Software, Department of Radiology, The First Affiliated Hospital of Dalian Medical University, Taiyuan, China; ^3^ Department of Radiology, Peking University First Hospital, Beijing, China; ^4^ Cardiovascular Department, Anshan Municipal Central Hospital, Anshan, China; ^5^ Department of Orthopaedics, The First Affiliated Hospital of Dalian Medical University, Dalian, China

**Keywords:** soft tissue sarcoma, multi-modal imaging, medical segmentation, self-supervised learning, medical image

## Abstract

**Introduction:**

Soft tissue sarcomas, similar in incidence to cervical and esophageal cancers, arise from various soft tissues like smooth muscle, fat, and fibrous tissue. Effective segmentation of sarcomas in imaging is crucial for accurate diagnosis.

**Methods:**

This study collected multi-modal MRI images from 45 patients with thigh soft tissue sarcoma, totaling 8,640 images. These images were annotated by clinicians to delineate the sarcoma regions, creating a comprehensive dataset. We developed a novel segmentation model based on the UNet framework, enhanced with residual networks and attention mechanisms for improved modality-specific information extraction. Additionally, self-supervised learning strategies were employed to optimize feature extraction capabilities of the encoders.

**Results:**

The new model demonstrated superior segmentation performance when using multi-modal MRI images compared to single-modal inputs. The effectiveness of the model in utilizing the created dataset was validated through various experimental setups, confirming the enhanced ability to characterize tumor regions across different modalities.

**Discussion:**

The integration of multi-modal MRI images and advanced machine learning techniques in our model significantly improves the segmentation of soft tissue sarcomas in thigh imaging. This advancement aids clinicians in better diagnosing and understanding the patient's condition, leveraging the strengths of different imaging modalities. Further studies could explore the application of these techniques to other types of soft tissue sarcomas and additional anatomical sites.

## 1 Introduction

Soft tissue sarcoma (STS) refers to sarcomas that occur in soft tissues such as fibrous tissue, striated muscle, smooth muscle, fat, and so on. Its incidence rate is similar to that of cervical cancer and esophageal cancer Gamboa et al. (1). Soft tissue sarcomas have complex components and a wide range of types, and they have different manifestations in clinical manifestations, medical imaging, and pathological characteristics Nystrom et al. (2). Soft tissue sarcomas may appear in various parts of the body, but the incidence rate of limbs is the highest, up to 40% - 50% Blay et al. (3). At present, the mainstream treatment plan for soft tissue sarcoma is mainly surgical resection and adjuvant treatment such as chemotherapy is needed when necessary Casali et al. (4). During the treatment of soft tissue sarcoma, it is important for clinicians to accurately identify the sarcoma area with the help of medical images to more accurately study and judge the patient’s condition and formulate a more stable clinical treatment plan. Because the imaging of soft tissue in CT is relatively close, it is not easy to distinguish between normal soft tissue and tumor areas, so MRI images are often used for imaging diagnosis of soft tissue sarcomas in clinical practice. Compared with CT, MRI images have higher soft tissue resolution, and MRI has more sequences, which can depict the shape size, internal signal strength, boundary texture, and tumor invasion of surrounding tissues from different angles and the information between different modes is complementary, it provides more judgment basis for detection of soft tissue tumor lesions, determination of origin, diagnosis of benign and malignant tumors, evaluation of invasion range and prediction of recurrence Chhabra et al. (5).

When using MRI images to diagnose soft tissue sarcomas, not only requires doctors to have high professional skills and attention, but also requires doctors to read a large number of MRI images, and comprehensively study and judge multiple modal MRI images of each patient. This method is not only time-consuming and laborious but also prone to missed and wrong judgments, what is more important is to judge whether the surrounding tissue of the soft tissue tumor area is tumor-induced edema or tumor infection, which has higher professional skill requirements for doctors. The development of deep learning makes it possible to diagnose the tumor area with the help of a computer on MRI images. The segmentation and detection of deep learning in lung nodes Bouget et al. (6), brain cancer segmentation Zhou et al. (7) Zhou et al. (8), liver cancer segmentation He et al. (9) and other tumor segmentation fields have been widely used. With the powerful feature extraction ability of the deep learning model, it can efficiently and accurately extract the tumor feature information in the MRI modal images of soft tissue sarcoma and automatically segment the sarcoma region.

In clinical practice, accurate diagnosis and treatment planning for soft tissue sarcomas are critical to patient prognosis. However, traditional diagnostic methods rely on physicians manually interpreting MRI images, which is not only time-consuming but also susceptible to subjective judgment, resulting in inadequate segmentation accuracy. In addition, the low contrast of soft tissues in CT imaging makes it more difficult to differentiate between tumor regions and normal tissues, further increasing the diagnostic complexity. To overcome these challenges, this study proposes a multimodal MRI image segmentation model based on self-supervised learning, aiming to improve the accuracy and efficiency of soft tissue sarcoma segmentation.

While current segmentation techniques have difficulty in striking a balance between processing speed and segmentation accuracy, our method not only improves the segmentation quality but also optimizes the computational efficiency by introducing an attention mechanism and a self-supervised learning strategy. By adaptively fusing features from different MRI modalities, our model can identify and segment tumor regions more accurately, which is crucial for accurate surgical planning and treatment decisions. In addition, considering the diversity and incompleteness of data in clinical practice, our self-supervised learning strategy effectively addresses the challenges posed by small sample sizes by enhancing the encoder’s feature extraction capability, improving the model’s generalization ability and robustness.

The contribution of this study is to provide a new assisted diagnosis model, which has the potential to significantly reduce the workload of physicians, improve the accuracy of clinical decision-making, improve patient outcomes, and provide new perspectives and solutions in the field of medical image analysis.

Our contributions in this paper are three-fold:

1. We collect multi-modal MRI image data from 50 patients with soft tissue sarcoma and obtain a multi-modal MRI image dataset of soft tissue sarcoma patients through a series of preprocessing operations.
2. We design a soft tissue sarcoma segmentation model and propose an attention mechanism to encourage the model to learn feature information from different modalities.
3. We propose a self-supervised training strategy to alleviate the problem of small data volume in our dataset.

**Figure 1:**
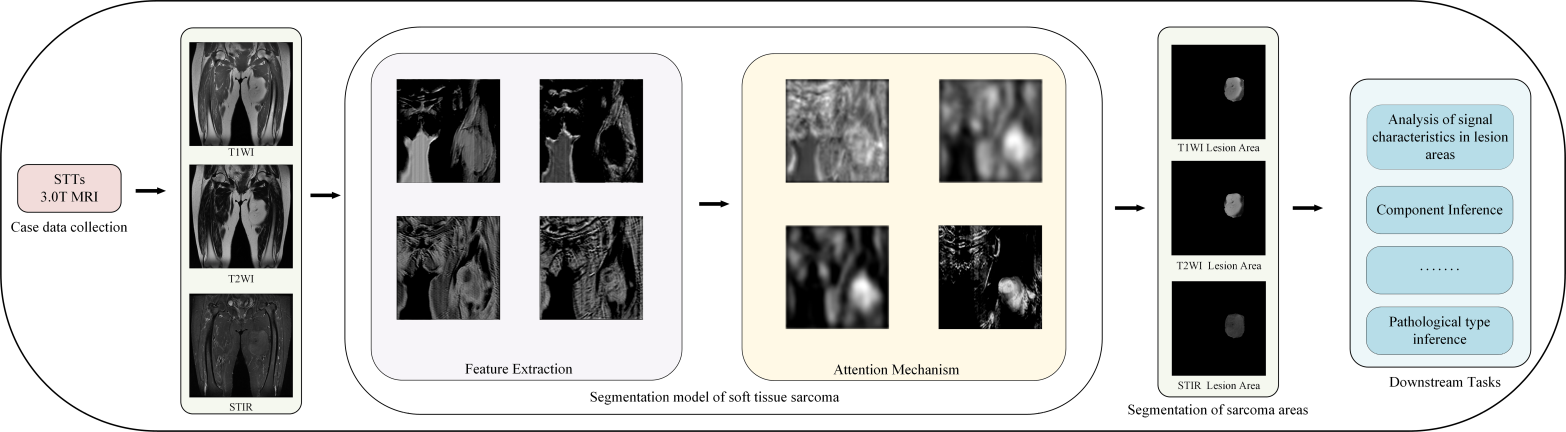
Our workflow.

**Figure 1** shows our workflow.

## 2 Relevant work

### 2.1 Segmentation of soft tissue sarcoma

At present, there are many deep-learning-based multimodal tumor segmentation methods Cao et al. (10) Tran et al. (11) Cheng et al. (12). Most of these methods are inspired by complete convolutional neural networks (FCN) Long et al. (13), one of the earliest attempts to use CNN in the direction of image segmentation. Its variant UNet Ronneberger et al. (14) model is a classical model to solve problems in the field of medical segmentation. The UNet network is an encoder-decoder structure. It uses skip connection to combine the low-level features and high-level semantic information of the same scale feature graph so that the model can better learn the local features and global features of the input image. Because UNet is simple, efficient, easy to understand, easy to construct, can be trained on small data sets, and has an outstanding segmentation effect, it is widely used in all directions of semantic segmentation. Inspired by UNet, Dolz et al. (15) proposed multimodal UNet suitable for multimodal medical image segmentation, and this network has been proven to be effective in multimodal medical image segmentation such as brain tumor segmentation of multimodal MRI image Mehta et al. (16) Myronenko (17) and lung tumor segmentation of PET/CT image Zhong et al. (18).

The main objective of the soft tissue sarcoma segmentation task is to label malignant tumors in soft tissues at the pixel level. Deep learning has been applied in the segmentation of soft tissue sarcoma. Zhong et al. (18) NSCLC tumors in PET-CT images are co-segmented by DFCN, and CT and PET information are considered at the same time. The network structure consisted of two coupled 3D-UNets to share complementary information between PET and CT. However, they do not take into account that each mode placed different emphasis on tumor characteristics, which affected tumor classification. Peng et al. (19) A deep multimodal collaborative learning is proposed and an end-to-end volumetric deep learning architecture is introduced to learn PET-CT complementary features. To distinguish different modes, a mode-specific sarcoma segmentation model is developed in reference Tang et al. (20) to realize multi-mode feature learning through the mode-specific encoder and decoder branches, and the use of resource-efficient densely-connected convolution layers.

### 2.2 Attention mechanism

The human eye will selectively pay more attention to the information of some regions while ignoring the information of other parts when observing things. This mechanism is called the attention mechanism. Literature Hu et al. (21) proposes that the SE module can explicitly establish the dependency between feature map channels adaptively generate feature map channel weights and recode the feature information in the feature map. Based on literature Hu et al. (21), literature Roy et al. (22) proposes three variants of the SE module, cSE, sSE, and scSE. Among them, cSE and SE module in document Hu et al. (21) both have the function of establishing the dependency between the channels of feature maps, and sSE module has the function of establishing the dependency between the spatial location information of feature maps, while scSE module parallels sSE module and cSE module to establish both the channel dependency between feature maps and the spatial dependency between feature maps. The experiment in document Roy et al. (22) shows that three variants are applied to the UNet model. In the challenge of MRI whole-brain segmentation, better segmentation performance of the original UNet can be achieved. Literature Woo et al. (23) proposes a simple and effective convolution attention module, which calculates the attention map along the two independent dimensions of input feature map channel and space, and then performs adaptive feature refinement according to the attention map and input feature map, which can be conveniently embedded in the convolution framework. Literature Oktay et al. (24) also proposes a new attention gate mechanism for medical imaging, and introduces it to the jump junction of the UNet structure. The image segmentation experiment is carried out on two large CT abdominal data sets, proving that the introduction of an attention gate mechanism to UNet can improve the sensitivity and prediction accuracy of the model.

## 3 The proposed data set

**Figure 2:**
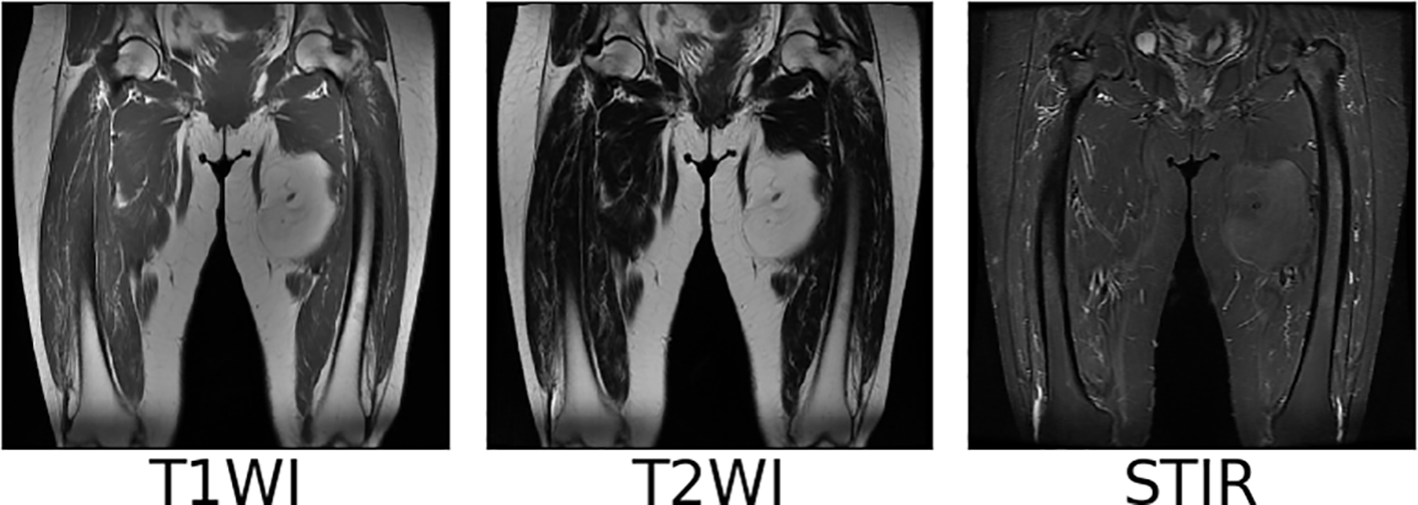
Examples of T1WI, T2WI and STIR image.

We collect coronal (COR) T1WI, T2WI, and STIR modal MRI images of 45 patients with soft tissue sarcoma at thigh sites from our hospital, as shown in **Figure 2**. The original images are preprocessed, and soft tissue sarcoma areas are jointly marked by multiple clinicians, after which cropped sectioning operations are performed on multiple modal MRI images of the same shot angle of the same patient, resulting in two multimodal imaging data sets of soft tissue sarcomas at thigh sites. Below is a detailed description of the preprocessing operations.

### 3.1 Resample

**Figure 3:**
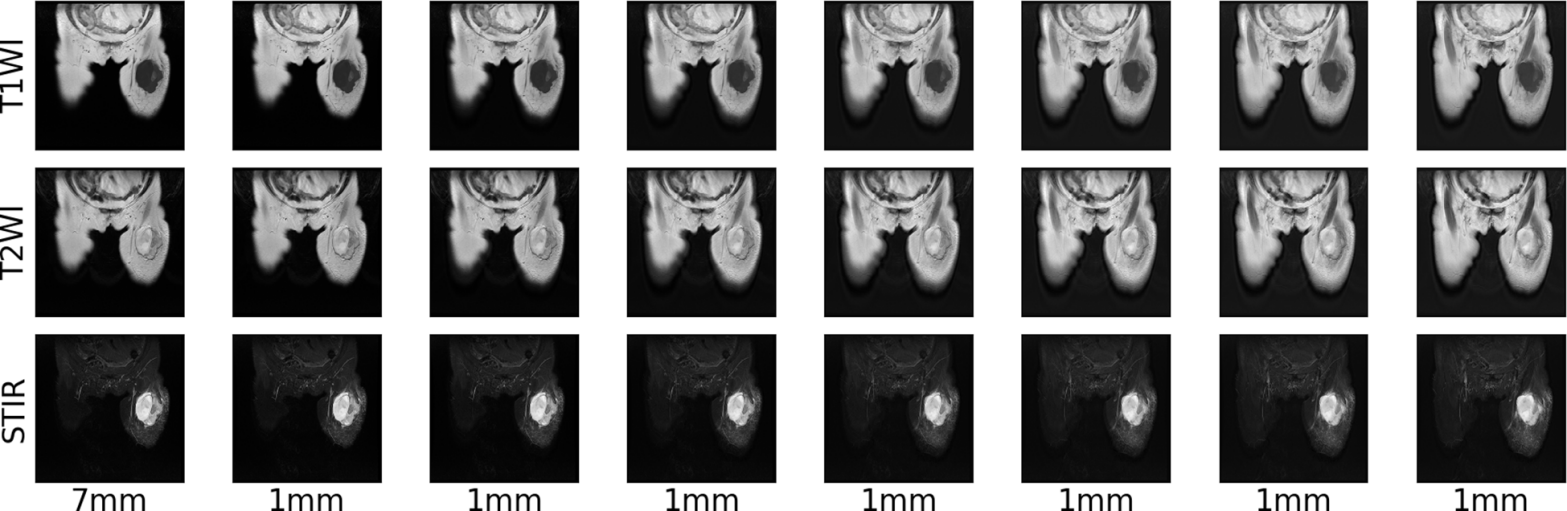
MRI resampling: 9 mm thickness of each modality of the original MRI image of a patient with a soft tissue sarcoma, 1 mm thickness of each modality of the MRI image after interpolation resampling.

Due to different machine models and shooting parameters, the original MRI image sequences of patients with soft tissue sarcoma have inconsistent pixel sizes and thickness and granularity of different scanning surfaces, which is not good for the training of the convolutional depth model. Therefore, we first use the interpolation algorithm to resample the original MRI images of patients with soft tissue sarcoma, so that the pixel size and the distance between each scanning surface in coronal and sagittal positions of all MRI images are 1mm, and the size of each scanning surface is 512*512, which not only reduces errors caused by data inconsistency, but also increases the number of MRI sequence sections. **Figure 3** shows the example of T2WI modality in MRI images of a patient with soft tissue sarcoma with an original thickness of 9mm before and after interpolation resampling.

### 3.2 Maximum-minimum normalization

Although MRI images are similar to CT images, each pixel on each scanning plane in different mode sequences represents the signal intensity of the corresponding position when shooting images, in the same mode sequence of MRI images, the sarcoma region of the same patient may show different intensity signals under different shooting parameter settings, this makes it impossible to use the commonly used window adjustment method in CT image processing to uniformly operate all MRI images of all patients to highlight the tumor area and solve the problem of inconsistent signal intensity caused by the setting of shooting parameters in RI images. Therefore, we choose to use the maximum-minimum operation for normalization.

Since the max-minimum normalization operation is prone to the influence of extreme values, before the normalization, we first remove the maximum and minimum 5% pixels of signal intensity in each scan surface and use the remaining 90% pixels of signal intensity for operation. The maximum-minimum normalization formula is shown in Equation 1. Where, *S_max_* and *S_min_* respectively represent the maximum and minimum values of signal intensity in 90% pixel points, and *S* and *S* ^′^ respectively represent the original signal intensity and normalized signal intensity at each position of each scanning surface.


(1) $$S^{\prime }=\frac{S-Smin}{Smax-Smin}v$$

**Figure 4:**
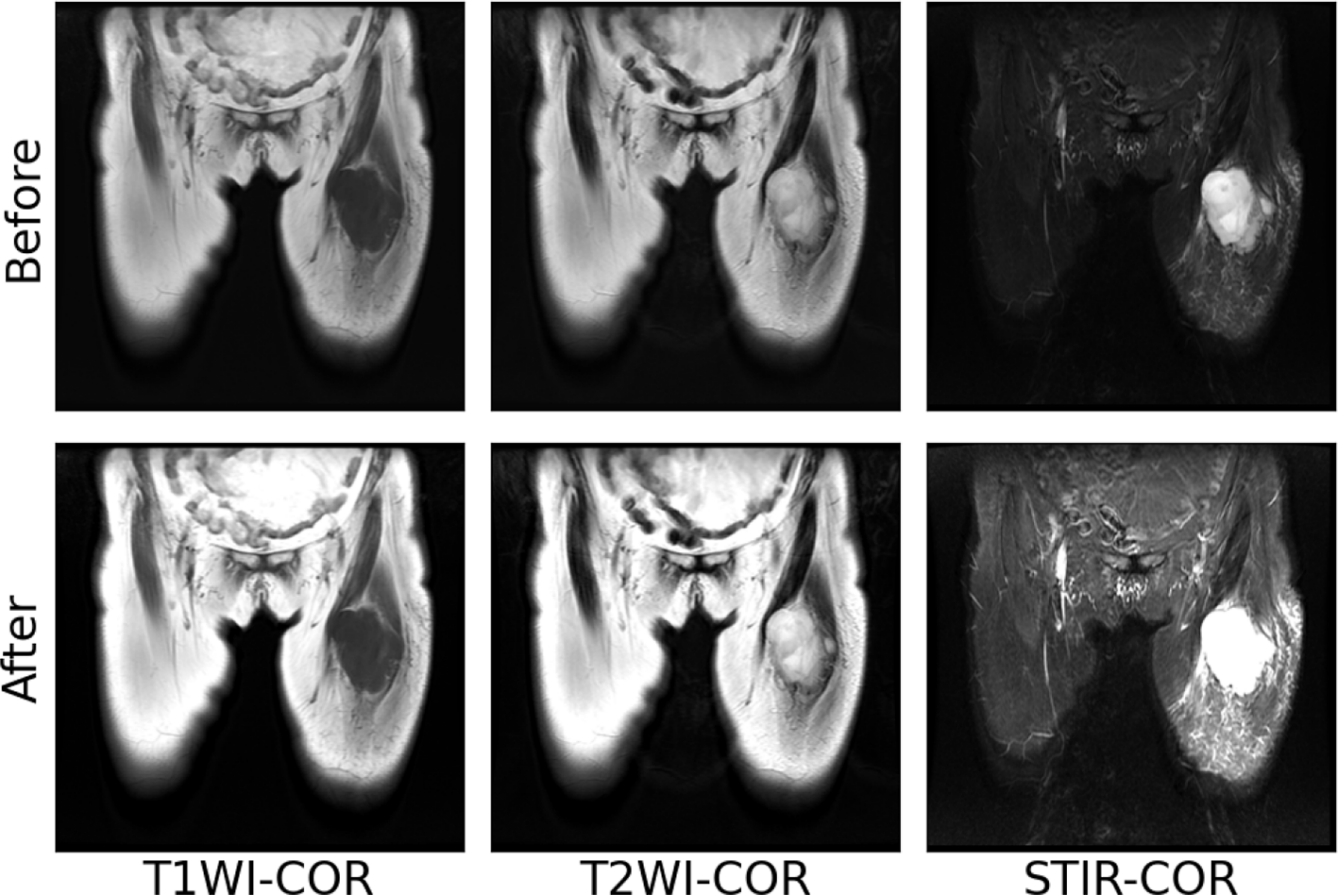
Maximum–minimum normalized pre-post images of individual modal sequences of MRI in patients with soft tissue sarcoma.

The images of T1WI, T2WI, and STIR modalities in coronal and sagittal positions of a patient with soft tissue sarcoma after the above normalization operation are shown in **Figure 4**. After maximum-minimum normalization, the contrast between the tumor area and other areas in each mode of soft tissue sarcoma is enhanced.

### 3.3 MRI imaging markers of soft tissue sarcoma

**Figure 5:**
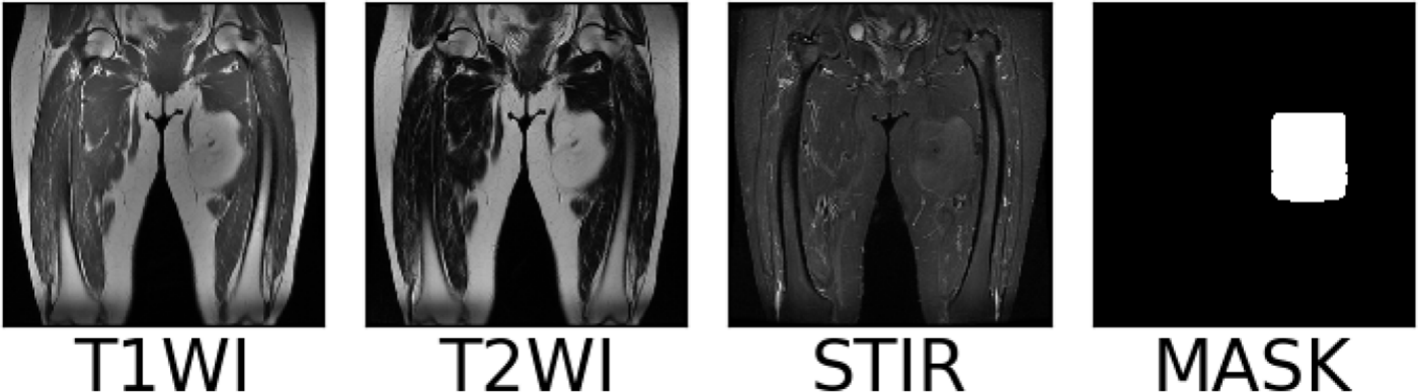
Box and mask images of sarcomatous areas in each modal series of soft tissue sarcomas.

Multiple clinicians use a rectangular box to mark the sarcoma region in the MRI images after the above preprocessing operations. At the same time, the coordinates of the upper left and lower right corner of the rectangular box are recorded in the array, and the same scanning surface of the MRI sequence of different modalities in the same patient shared a marker box. The MRI images, marker frame, and mask images of a soft tissue sarcoma patient in each modality are shown in **Figure 5**, in which the red rectangular box is the marker frame, and from left to right are T1WI modality, T2WI modality, STIR modality, and mask image.

### 3.4 Tumor area cutting and sectioning

**Figure 6:**
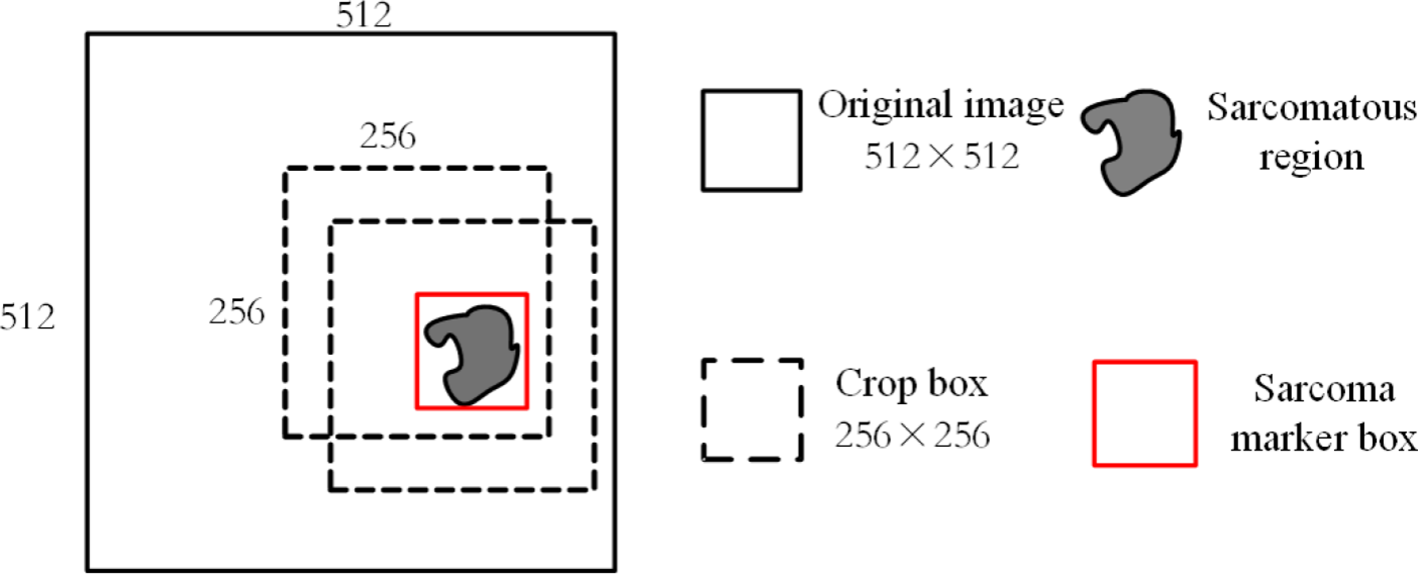
Schematic diagram of tumor region cropping operations.

It requires a lot of computational resources to use the 512*512 MRI modal images obtained by the above processing as input. At the same time, as the average proportion of soft tissue sarcoma area in each scanning surface of the image is about $\frac{1}{16}$ , we use a 256*256 cropping box around the marker box of the sarcoma area for MRI during training. The image sequence is clipped so that the area clipped by the clipping box not only includes all the areas marked by the tumor marker box but also does not intercept the parts outside the original area of each scanning surface, as shown in **Figure 6**. At the same time, each modal sequence of MRI is sliced and 64 scanning surfaces in the middle of each sequence are taken. The same pruning and sectioning operations are performed on all MRI modal sequences of the same patient to obtain 64×256×256 aligned multi-modal MRI images of soft tissue sarcoma.

## 4 Model

### 4.1 Network structure

**Figure 7:**
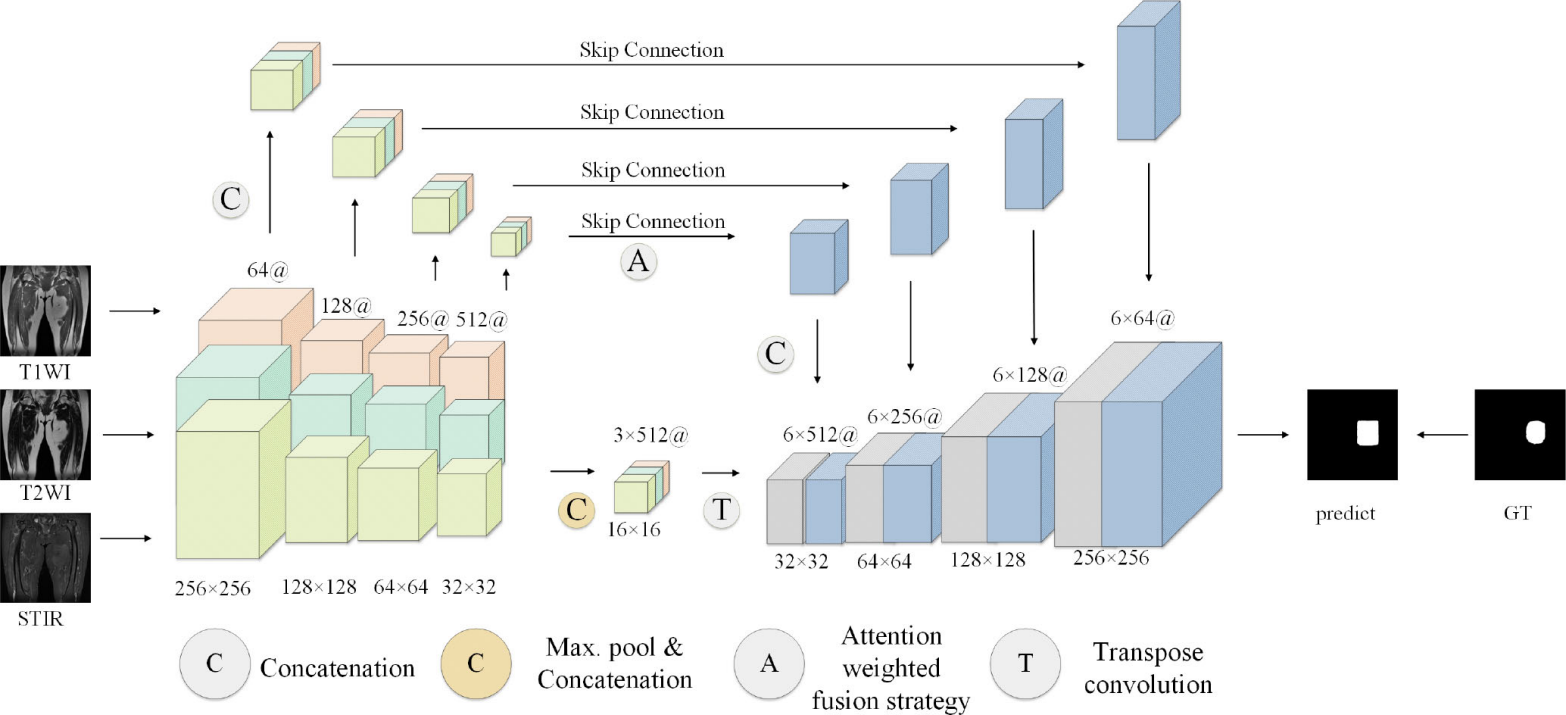
Overall structure diagram of the model.

The proposed model generally follows the encoder-decoder structure, but the difference is that encoders have multiple encoding paths to accept multi-modal inputs. This is shown in **Figure 7**, the model encoding ends have three coding paths in the same structure. Each coding path has 4 layers, and each layer is composed of two consecutive convolution operations and one downsampling operation. The size of the convolution kernel is 3 ∗ 3, the number of convolution kernels is doubled layer by layer from 64, and the number of them in the last layer is 512. The downsampling operation is composed of a 2 ∗ 2 pooling layer. Between the encoding path and the decoding path is the attention fusion strategy module, which fuses all modal-related features from the coding path to better extract features. The fused features are transmitted to the decoding end. The decoding end is also composed of 4 layers, each layer is composed of two consecutive 3 ∗ 3 convolution and 2*2 upsampling layers. Each layer of the decoding end and each layer of the coding-end correspond to each other, and the corresponding layers are transferred to the decoding end through a hop connection. The features obtained by the decoding end pass through a layer of Softmax layer to obtain the classification of each pixel and finally output the prediction results of the model.

### 4.2 Feature fusion

**Figure 8:**
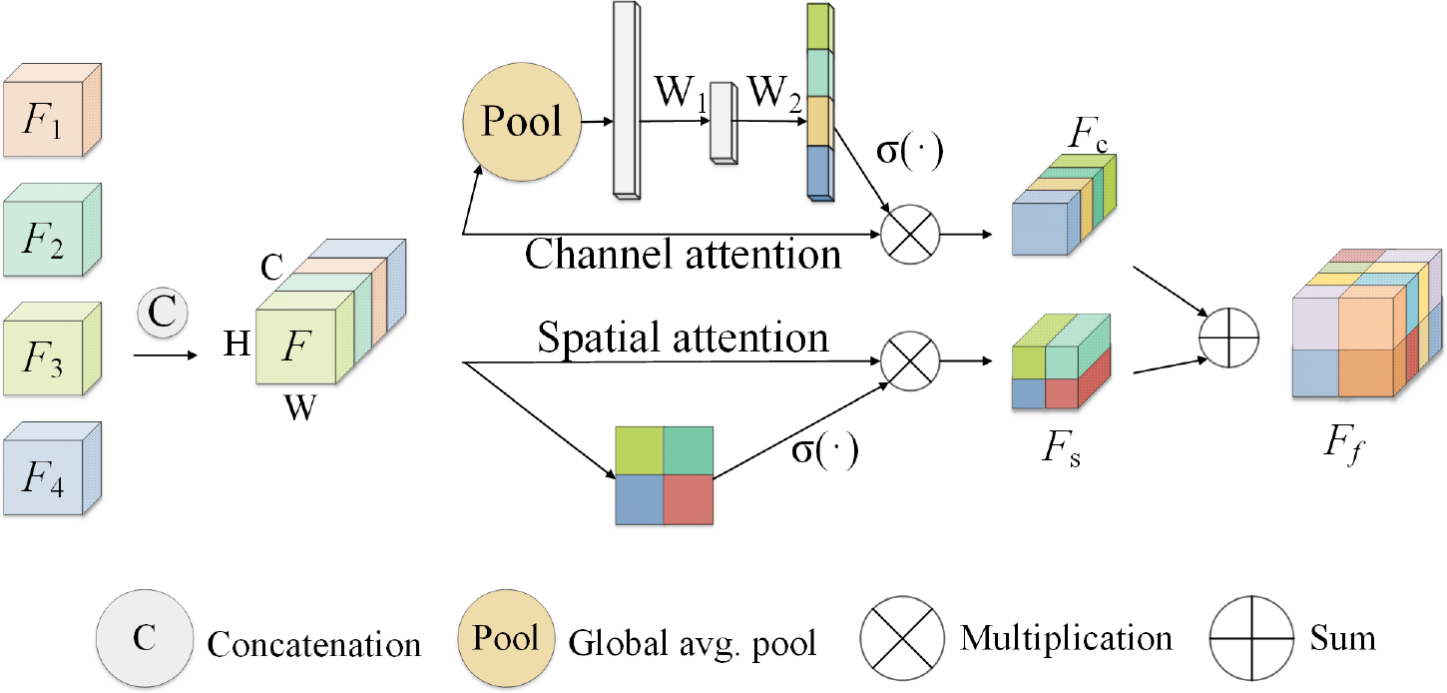
Attention-weighted fusion strategy. The input feature map is (expressed as *F*_1_, *F*_2_, *F*_3_, *F*_4_) processed by the channel attention mechanism and the spatial attention mechanism to obtain the new feature maps *F_c_* and *F_s_*, and the modal adaptive attention weighted feature map *F_f_* is obtained after fusing *F_c_* and *F_s_*.

Considering that the signal intensity of soft tissue sarcoma in the T1WI modality is weaker than that of other tissues, the signal of the tumor region in the T2WI and STIR modalities is stronger than that of other tissues, and the coding path of each mode of MRI image extracts the feature map of the corresponding modality and makes a simple connection that cannot effectively use the feature information from different modalities, we propose a new attention weighted fusion strategy to fuse the characteristics of soft tissue sarcoma regions with different modalities. Its structure is shown in **Figure 8**. We use *F*_1_, *F*_2_, *F*_3_, and *F*_4_ to represent the feature information of different modalities extracted by the coding end, and stitch the feature information of different modalities along the channel direction to obtain the feature map *F*, the feature map *F* passes through the channel attention mechanism module and the spatial attention mechanism module successively to obtain a new feature map *F_c_* and *F_s_*, and add *F_c_* and *F_s_* to obtain the fusion feature map after modal adaptive attention weighting *F_f_*.

In channel attention mechanism in the module, characteristic figure from different code paths together without $F\in R^{H}{\;}^{*W*C}$ is regarded as $U=\;\left[u_{1},u_{2},\ldots ,u_{C}\right]$ feature vector, where $u_{k}\in R^{H}{\;}^{*W},i\in \;1,2,\ldots ,C$ gets the *k* vector element of the vector $Z\in R^{1*1*C}$ after the global pooling operation. The above process is shown in Equation 2.


(2) $$Z_{k}=\frac{1}{H*W}\sum_{i}^{H}\sum_{j}^{W}u_{k}\left(i,j\right)$$

After the global average pooling operation, the spatial information in the feature map is mapped to the feature vector *Z*. This vector passes through two fully connected layers and is transformed to $\hat{Z}=W_{2}\left(\delta \left(W_{1}*Z\right)\right)$ , where $W_{1}\in R^{\frac{C}{2}*C},W_{2}\in R^{C*\frac{C}{2}}$ is the weight of the two fully connected layers, *δ*() is the ReLU activation function. Then activate the function via Sigma (*σ*()). Map it between the interval [0,1] to obtain the channel attention weight of the special graph *F*, and multiply this weight with the feature map *F* to obtain the feature map weighted by the channel attention mechanism *F_c_*. The above process is shown in Equation 3.


(3) $$F_{c}=\left[\sigma \left(\hat{z_{1}}\right)u_{1},\sigma \left(\hat{z_{1}}\right)u_{1},\ldots ,\sigma \left(\hat{z_{C}}\right)u_{C}\right]$$

In the spatial attention mechanism module, the feature map $F\in R^{H}{\;}^{*W*C}$ is seen as $U\;=\left[u(1,1),u(1,2),\ldots ,u(i,j)\ldots ,u(H,W)\right]$ spliced from different coding paths is regarded as the feature vector of $U=\left[u(1,1),u(1,2),\ldots ,u(i,j)\ldots ,u(H,W)\right]$ , where $u\left(i,j\right)\;\in R^{1*1*C},i\in \;1,2,\ldots ,H,j\in \;1,2,\ldots ,W$ is regarded as the feature component of the feature map at (i, j) spatial position. When using spatial attention to weight the feature map, the convolution operation with the weight $W_{s}\in R^{1*1*C*1}$ is used to map the vector U to obtain the vector $q\in R^{H}{\;}^{*W}$ , and the vector $q^{i,j}$ represents the linear combination representation of the channel direction features on the spatial position (i,j) of the feature map. Then, the maximum pooling operation is used in the channel direction to obtain the vector $p\in R^{H}{\;}^{*W}$ , and the vector $p^{i,j}$ represents the most significant feature in the channel direction on the spatial position (i, j) of the feature map. After adding the vectors p and q, use the Sigma activation function to scale to [0, 1], and multiply with the input feature map to obtain a spatially weighted attention feature map *F_s_*. The above process is shown in Equation 4.


(4) $$F_{s}=\left[\sigma (p^{1,1}+q^{1,1})u^{1,1},\ldots ,\sigma (p^{H,W}+q^{H,W})u^{H,W}\right]$$

The channel-weighted feature map *F_c_* and the spatially weighted special map *F_s_* are added and fused to obtain the attention-weighted fusion feature representation *F_f_* of the input feature map. The above process is shown in Equation 5.


(5) $$F_{f}=F_{c}+F_{s}$$

This method can be applied to the combination of different modal inputs and can extract more important semantic features in the channel direction and spatial direction, and better fuse the feature information from different modalities.

### 4.3 Self-supervision training

MRI images from different modalities provide different descriptions of the same soft tissue sarcoma site from different perspectives, which makes multi-modal MRI images provide more information about the tumor site than single-modality MRI images, but the amount of multi-modal MRI image data collected for training our model is still lacking. Therefore, we choose to use the self-supervised learning strategy to overcome the problem of insufficient network training due to the small amount of data. Although self-supervised learning strategies can also be realized by rotation, scaling, inversion, and other means, considering that when patients with soft tissue sarcoma in the thigh region take MRI, the relative position of the entire thigh in each modality of the MRI image is almost the same, and the alignment between different modalities is required after using rotation, zoom and other operations, so we design a masking task for self-supervised learning based on the literature Fang et al. (25). As shown in Algorithm 1, we present the training algorithm of the model.

**Algorithm 1:** Calculation of the model during training.

Input: image of size 3 × 256 × 256 Initialization: *modality* ← 3*, layer* ← 4*, F*[*modality*][*layer*] *x* ← tensor of image $x^{\prime }\leftarrow$ tensor of image with mask //The process of Encoder **for** *t* refers to $x,x^{\prime }$ **do** **for** *m* ← 0 to *modality* − 1 **do** **for** *l* ← 0 to *layer* − 1 **do** *t*[*m*] ← *Conv_Encoder_ *[*m*][*l*](*t* [*m*]) *F*[*m*][*l*] ← *t*[*m*] *t*[*m*] ← *MaxPool*(*t*[*m*]) **end for** **end for** **end for** *y* ← *Conv_Bottleneck_ *(*x*) //The process of Decoder **for** *l* ← *layer* − 1 to 0 **do** *y* ← *TransConv*[*l*](*y*) *y* ← [*y*;*FeatureFusion*(*F*[]:[*l*])] *y* ← *Conv_Decoder_ *[*l*](*y*) **end for** *y* ← *EndConv* (*y*) Output: *x*, *x′*, *y*

**Figure 9:**
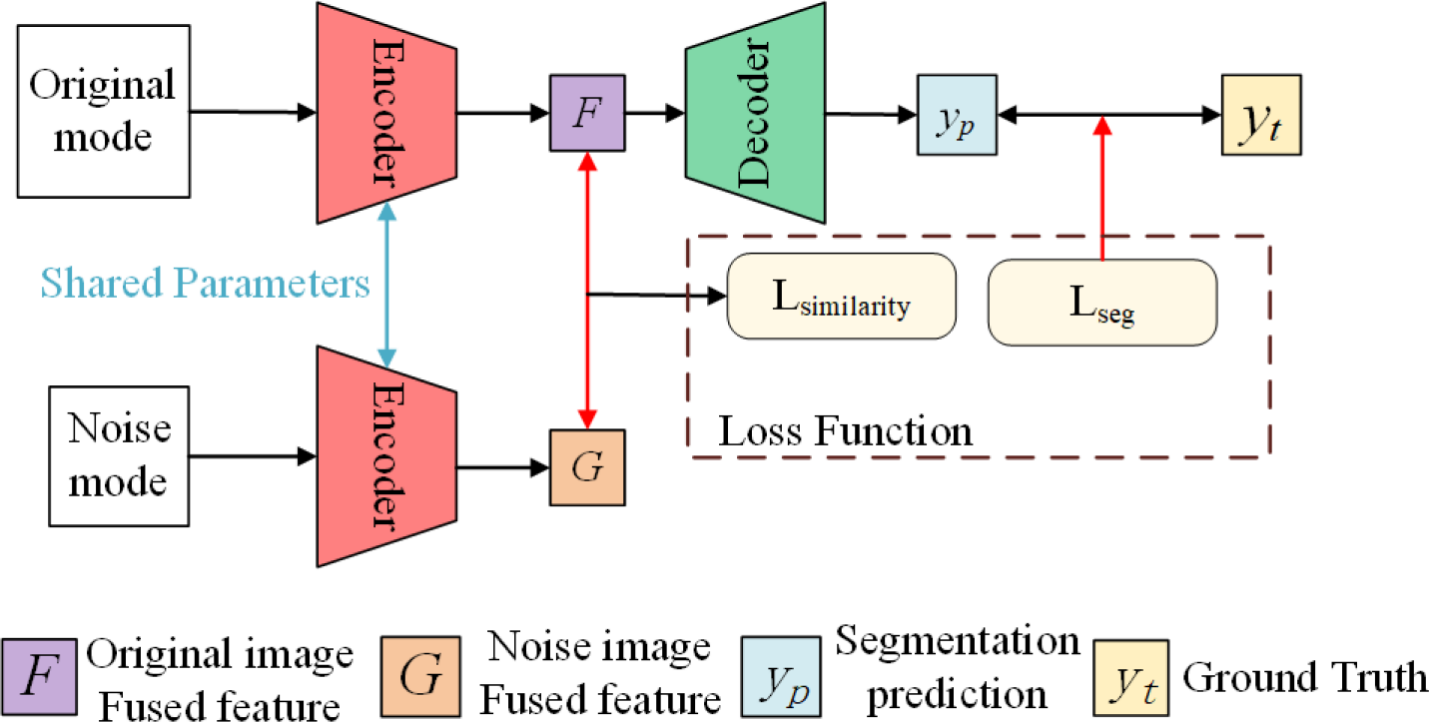
Self-supervised learning strategies.


As shown in **Figure 9**, our self-supervised learning uses two coding ends that share weights, and the structure of these two coding ends is consistent with each coding path of the multi-coding end as described earlier. The upper branch is used to perform the segmentation task of single-modal image as input. The lower branch is only used to perform the feature extraction task of single-modal image with mask noise as input, and shares parameters with the upper branch for training. In detail, for the input *I* of each modality, we randomly shield the border of the sarcoma region with a mask of 30*30 at the boundary of its tumor region and obtain the noisy input $I^{'}.I$ encoded by the upper branch encoder to obtain the feature $\vec{F},\;I^{'}$ to obtain the feature $\vec{G}$ by the lower branch encoder encoding. Bouget et al. (6), we use cosine similarity *L_Similarity_* and segmentation loss *L_Dice_* to guide the coding-end to perform feature extraction on single-modal images, to strengthen the ability of the coding path corresponding to each modal input to extract features. The above process is shown in Equations 6–7, and the total loss function is shown in Equation 8. The total loss function for each modal coding path reinforced using self-supervised learning is:

(6) $$L_{Similarity}=\frac{\vec{F}*\vec{G}}{\|\vec{F}{\|}_{2}*\|\vec{G}{\|}_{2}}$$

(7) $$L_{Dice}=-\frac{2\sum y_{p}y_{t}}{\sum y_{p}+\sum y_{t}}$$

(8) $$L_{self-sup}=L_{Similarity}+L_{Dice}$$

## 5 Experimental setup

### 5.1 Implementation details

We randomly divide the two datasets into 80% train sets and 20% test sets, use PyTorch 1.10 to implement our network, and train 100 epochs on NVIDIA GeForce GTX 3090. We optimize our network with Adam Optimizer. The learning rate of the optimizer is 1*e* − 5 and if the loss is verified not improved within 10 epochs, then we will terminate the training in advance to avoid overfitting. The choice of learning rate is based on experimentation and experience. In this study, the learning rate of the optimizer is a common choice as it usually provides stable and efficient performance during training, thus helping the model to converge to a better solution.

### 5.2 Evaluation indicators

Each pixel point of each scanned surface in different models of the soft tissue sarcoma MRI is one of the two categories of tumor areas or non-tumor areas. We use the Dice coefficient, sensitivity, specificity, and Hausdorff distance to evaluate the performance of the model. The calculation formula of the evaluation index is shown in Equations 9–12, of which *TP*, *FP*, *TN*, and *FN* represent the number of pixels of true positive, false positive, true negative, and false negatives, respectively. And *d*(*x,y*) represents the Euclidean distance between *x* and *y*.


(9) $$Dice=\frac{2TP}{2TP+FN+FP}$$

(10) $$Sensitivity=\frac{2TP}{TP+FN}$$

(11) $$Specificity=\frac{TP}{TN+FP}$$

(12) $$Hausdor\;f\;f=max\left\{d\left(y,y^{\prime }\right),d\left(y^{\prime },y\right)\right\}$$

### 5.3 Experiment results

**Figure 10:**
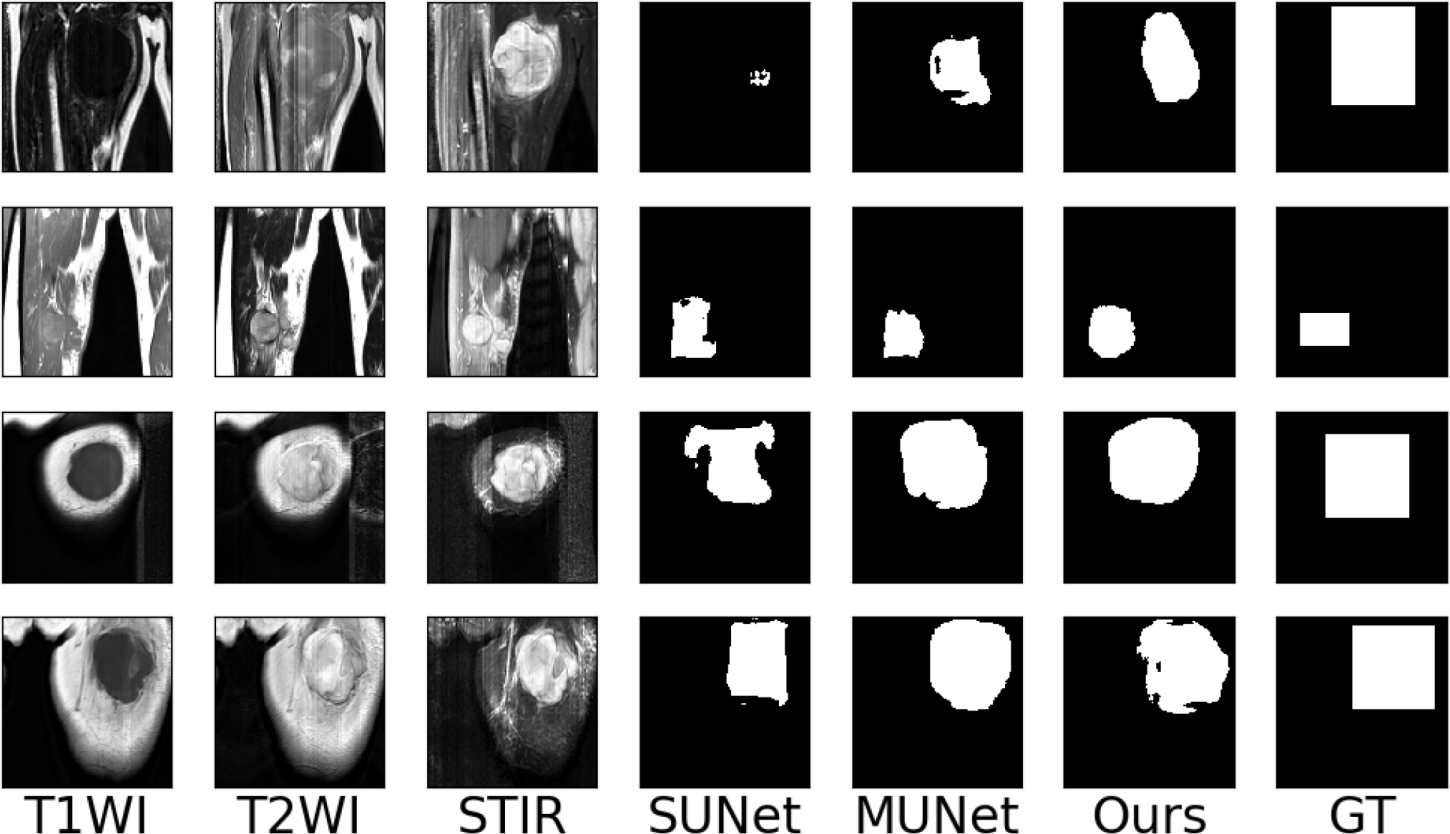
Examples of the segmentation results on our dataset. (SUNet) Single encoder UNet, (MUNet) Multi encoder UNet, (GT) Ground Truth.

To make the experimental results more realistic and reliable, we use the UNet network as the backbone to set up a single-encoder UNet model and a multi-encoder UNet model. The settings of the number of convolution cores and the convolution size of each layer of the two models are consistent with our model. The experimental results are shown in **Table 1**. It can be seen that our models are far beyond the single-encoder UNet and ordinary multi-encoder in the Dice score and sensitivity, which shows that our model’s dividing accuracy and robustness are better than the two models above. And our model is not much different from the highest score in pixel accuracy and Hausdorff distance, which also illustrates the superiority of our model. In **Figure 10**, we show the experiment results that we use different models to segment the same patient’s sarcoma. It can be seen from the figure that our model is better on the boundary segmentation of tumors.

**Table 1:** Segmentation results of different modal. SUNet represents single-encoder UNet, and MUNet represents multi-encoder UNet.

Model	Acc	Dice	Sensitivity	Specificity	Hausdorff95
SUNet	0.8875	0.4425	0.4379	0.9362	60.8418
MUNet	0.9301	0.4899	0.4693	0.9828	26.5417
Ours	0.9242	0.5430	0.5258	0.9829	33.7487

## 6 Discussion

Although currently we have collected the MRI multi-modal image data of 45 patients with soft tissue sarcoma at the thigh, made relevant data sets, and designed experiments to verify the effectiveness and usability of the data sets we have made, it is important to further study the pathogenesis of soft tissue sarcoma and design a better computer-aided system to help clinical doctors diagnose. The amount of data in our dataset is still insufficient. At the same time, in clinical diagnosis, patients may be missing a certain modality in the MRI image due to various reasons. We can also design experimental research on tumor segmentation algorithms in the case of modality loss in the MRI image of soft tissue sarcoma based on our dataset, and design the strategy of modality fusion in the case of modality loss. At the same time, we will also study the modal completion method in the absence of modal in future work.

## Data availability statement

The original contributions presented in the study are included in the article/supplementary materials, further inquiries can be directed to the corresponding author.

## Author contributions

MZ: Conceptualization, Methodology, Investigation, Formal analysis, Writing – original draft. CG: Data curation, Writing – original draft. YZ: Visualization, Investigation. XG: Resources, Supervision. CF: Validation. SW: Writing – review & editing, Methodology, Investigation, Funding acquisition.
